# Understanding the clinical care pathway of patients with mild cognitive impairment due to Alzheimer's disease and dementia due to Alzheimer's disease: Results from a global real‐world survey

**DOI:** 10.1002/alz70858_100805

**Published:** 2025-12-25

**Authors:** Sharon Cohen, Sarah Cotton, Juan Fortea, Hisatomo Kowa, Nachiappan KR, Luis R Solís Tarazona, Chloe Walker

**Affiliations:** ^1^ Toronto Memory Program, Toronto, ON, Canada; ^2^ Adelphi Real World, Bollington, United Kingdom; ^3^ Memory Unit, Hospital de Sant Pau, Barcelona, Spain; ^4^ School of Health Sciences, Kobe University, Hyogo, Japan; ^5^ Novo Nordisk GBS, Bengaluru, India; ^6^ Novo Nordisk A/S, Søborg, Denmark

## Abstract

**Background:**

With the emergence of disease modifying treatments, the importance of early diagnosis of mild cognitive impairment (MCI) and mild dementia due to Alzheimer's disease (AD) has increased. This research explores the global clinical care pathway with the aim of highlighting areas for improvement for early and accurate diagnosis.

**Method:**

Data were drawn from the Adelphi Real World AD Disease Specific Programme™, a cross‐sectional survey of physicians and their patients in Canada, France, Germany, Italy, Spain, the United Kingdom, Japan, and the United States between December 2022 ‐ March 2024. Physicians reported data on patient's diagnostic pathway, Mini‐Mental State Examination (MMSE) score at first consultation, and treatment for approximately their next nine consulting patients diagnosed with MCI due to AD or dementia due to AD (clinically diagnosed or biomarker confirmed). Patients self‐reported their reasons for delaying consultation. Data was grouped by MMSE. Analyses were descriptive.

**Result:**

Overall, 829 physicians reported data for 5654 patients; 726 self‐completed forms. Patient mean (standard deviation) age was 76.7 (8.4) years and 48.0% were male. Of those with an MMSE score at first consultation, 20.6% had 26‐30, 47.2% 21‐25, 29.9% 11‐20, and 2.3% 0‐10. Time from symptom onset to first consultation was a median [interquartile range] of 20.7 [4.6, 52.0] weeks. Patients reported delaying first visiting a physician (75.1%), mainly due to believing their memory problems were a part of normal ageing (72.3%). Most patients first consulted a primary care physician (73.4% of patients with an MMSE of 26‐30; 58.7% 0‐10), of which 72.4% and 45.5%, respectively, were referred on for diagnosis. Key diagnostic tools were behavioural/cognitive assessments (91.8%), feedback from patient/patient's family (89.3%), and non‐AD specific blood tests (88.7%). Biomarker testing was infrequent (11.8%). When treatment was prescribed, lack of efficacy was the key driver for change in initial treatment (48.9%).

**Conclusion:**

Our results highlight a lack of awareness of early symptoms, inconsistent referral, and infrequent utilization of AD specific biomarkers. Addressing these challenges is pivotal to facilitate early, accurate diagnosis and intervention. Lack of efficacy was the main reason for changing initial treatment, indicating the need for better treatment options.

